# Effect of Thermal Exposure on Microstructure Evolution and Mechanical Properties of TC25G Alloy

**DOI:** 10.3390/ma16124462

**Published:** 2023-06-19

**Authors:** Zhuomeng Liu, Shewei Xin, Yongqing Zhao, Peiliang Zhu, Bohao Dang, Siyuan Zhang, Wei Zhou

**Affiliations:** 1 School of Materials Science and Engineering, Northeastern University, Shenyang 110819, China; lzm1227@yeah.net (Z.L.); zhuplchangan@163.com (P.Z.); 2 Northwest Institute for Nonferrous Metal Research, Xi’an 710016, China; doublehoon@163.com (B.D.); zsy314@msn.com (S.Z.); zhouwei2002563@163.com (W.Z.); 3 School of Materials Science and Engineering, Xi’an University of Architecture and Technology, Xi’an 710055, China

**Keywords:** TC25G alloy, thermal exposure, silicide, α_2_ phase, mechanical properties

## Abstract

The microstructure and room temperature tensile properties of heat-treated TC25G alloy after thermal exposure were investigated. The results show that the α_2_ phase dispersed in the α phase, and silicide precipitated firstly at the α/β phase boundary and then at the dislocation of the α_p_ phase and on the β phase. When thermal exposure was 0–10 h at 550 °C and 600 °C, the decrease of alloy strength was mainly due to the dominant effect of dislocations recovery. With the rise and extension of thermal exposure temperature and time, the increasing quantity and size of precipitates played an important role in the improvement of alloy strength. When thermal exposure temperature rose to 650 °C, the strength was always lower than that of heat-treated alloy. However, since the decreasing rate of solid solution strengthening was smaller than the increasing rate of dispersion strengthening, alloy still showed an increasing trend in the range of 5–100 h. When thermal exposure time was 100–500 h, the size of the α_2_ phase increased from the critical value of 3 nm to 6 nm, and the interaction between the moving dislocations and the α_2_ phase changed from the cutting mechanism to the by-pass mechanism (Orowan mechanism), and thus alloy strength decreased rapidly.

## 1. Introduction

The development of the modern aviation and aerospace industry requires a greater thrust to weight ratio and higher thermal efficiency of engines. Due to its excellent properties of light weight, high temperature resistance, and high strength and toughness, titanium alloy has been widely used for compressor blades, rotor blades, casings, and other components of aircraft engines [1,2,3,4]. The development demand of high performance aero-engines has driven the development of high temperature titanium alloy. At present, most titanium alloys working for long periods of time at high temperature are near-α titanium alloys [5,6,7] and α + β titanium alloys [8,9,10], and they are mainly Ti-Al-Sn-Zr-Mo-Si alloys. However, on account of the lack of stability of strength and microstructure at high temperature, its long-term use temperature cannot exceed 600 °C. Therefore, it is the study focus to explore the influence of microstructure evolution law on the mechanical properties of titanium alloys at high temperature environments for researchers who work in the Ti community.

During long-term service in high temperature environments, the main factors affecting the strength and ductility of Ti-Al-Sn-Zr-Mo-Si alloys are the decomposition of the residual β phase [11], the sequential transformation of the α phase to α_2_ phase (Ti_3_Al phase) [12], and the precipitation of silicide [13,14]. Wang and Chen et al. [15,16] noted that a large number of nanoscale needle-like secondary α phase precipitated from residual metastable β phase can effectively prevent the slip of dislocations. Zhang et al. [17] showed that the α_2_ phase gradually increased with aging temperature and time. When the grain size was increased to 15 nm, the interaction between the α_2_ phase and dislocations changed from the cutting mechanism to the by-pass mechanism, and the plasticity decreased sharply. M. Jayaprakash et al. [18] showed that the precipitation of (Ti, Zr)_x_Si_y_ was the main reason for improving the yield strength of alloys. A large number of experimental results [19,20,21] showed that the evolution rules of the decomposition of the residual β phase and the precipitate of the α_2_ phase and silicide are quite complex. In studies [22,23] of comprehensive mechanical properties of alloys, researchers hoped to form a small, dispersed, and uniform second phase during aging so as to improve the strength of alloys. When the influence of surface factors is not taken into account, the essence of the thermal exposure process is also an aging process [24].

At present, the maximum working temperature of TC25G (Ti-6.5Al-1.8Sn-4Zr-4Mo-1W-0.2Si) alloy is 550 °C, but no research group has systematically studied the effect of microstructure evolution on its mechanical properties after thermal exposure at 550 °C and above for different time periods. Therefore, this paper mainly conducted thermal exposure experiments on TC25G alloy at 550 °C, 600 °C, and 650 °C with different times and tested the tensile properties of alloy at room temperature. By systematically studying the microstructure evolution law of TC25G alloy after thermal exposure, the influence of precipitate precipitation behavior and the strengthening mechanism on the mechanical properties of alloy was discussed in order to provide sufficient data support for the practical application of TC25G alloy and also to provide reference for the research of other high temperature titanium alloys.

## 2. Experimental Procedure

**Figure 1:**
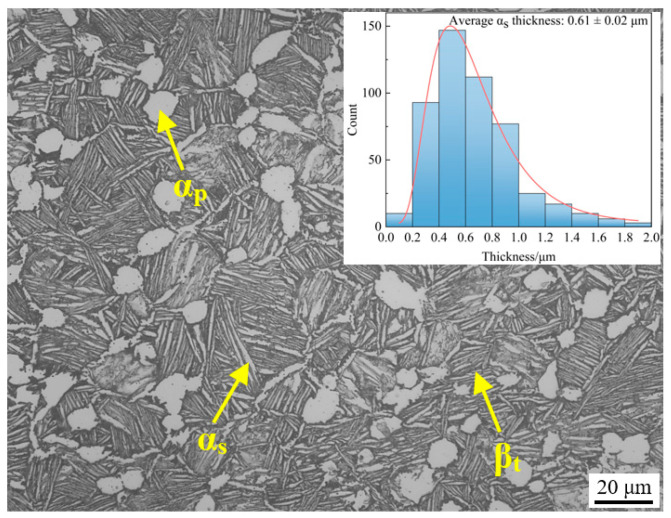
OM microstructure of TC25G alloy after heat treatment.

The TC25G alloy used in this study was provided by Northwest Institute for Nonferrous Metal Research, and the alloy was forged with optimal technology. Differential scanning calorimetry (DSC) was used to measure the (α + β)/β phase transition point temperature of TC25G alloy, which was found to be 972 ± 5 ℃. Then the optimal heat treatment process was formulated according to the comprehensive properties of alloy: 950 °C/3 h, AC + 580 °C/6 h, AC. Heat treatment samples with lengths of 67 mm and diameters of 12 mm were obtained from forging by an electro-discharged machine. The microstructure of alloy after heat treatment was a uniform bimodal microstructure, as shown in Figure 1. The volume fraction of the primary α phase (α_p_ phase) was 15%, the average diameter was 17 μm, and the volume fraction of the β transition structure (β_t_) was 85%. The average diameter of the secondary α phase (α_s_ phase) was 0.61 ± 0.02 μm.

Thermal exposure tests were carried out at 550 °C, 600 °C, and 650 °C, and the thermal exposure times were 5 h, 10 h, 20 h, 50 h, 100 h, 200 h, and 500 h. Samples were processed into standard tensile samples with lengths of 64 mm and diameters of 10 mm, and then the mechanical properties were finally tested at room temperature (i.e., thermal exposure mechanical properties were those without oxidized surfaces). The tensile tests were carried out on an Instro-1185 tensile machine in accordance with the ISO 6892 standard, and the strain rate was 2.5 × 10^−4^/s.

The microstructure of alloy was characterized by optical microscopy (OM, LEICADM 4000M, Wetzlar, Germany). Before analysis, samples were rough-ground, fine-ground, rough-polished, and fine-polished firstly and then etched with reagents (hydrofluoric acid:nitric acid:water = 1:3:7). An X-ray diffractometer (XRD, PW1700, Saarbrucken, Germany) was used for phase analysis. In addition, in order to better understand the morphology, size, quantity, and distribution of the precipitated phase after thermal exposure, a transmission electron microscope (TEM, FEI Tecnai G2 F20, Hillsboro, OR, USA) was mainly used to observe samples. Before TEM analysis, samples were ground to a thickness of less than 50 μm by hand grinding and then punched to obtain thin slices, each with a thickness of 50 μm and a diameter of 3 mm. Finally, TEM test samples were obtained by double spray dilution (perchloric acid:n-butanol:methanol = 1:7:12) in a double-jet electrochemical polishing machine (30 V, −30 °C). During TEM characterization, the acceleration voltage was controlled at 200 KV.

## 3. Results

### 3.1. Tensile Properties

**Figure 2:**
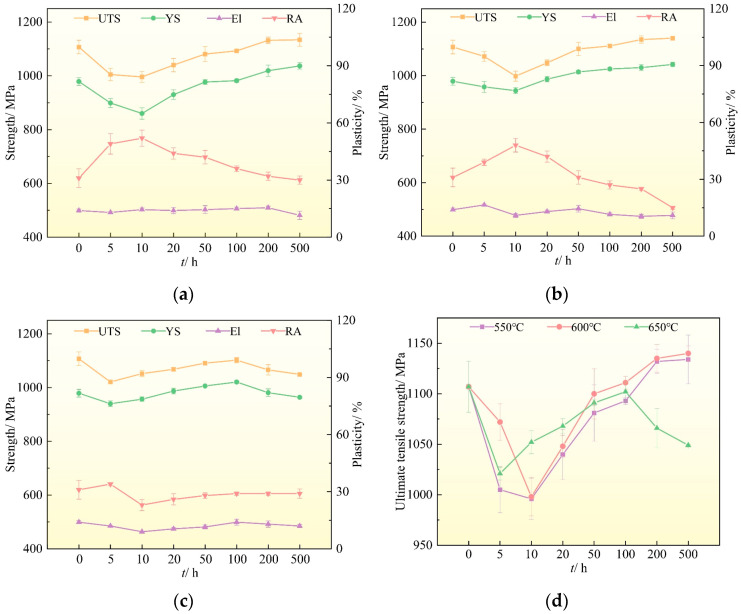
The effect of thermal exposure on the room temperature tensile properties of TC25G alloy: (**a**) 550 °C; (**b**) 600 °C; (**c**) 650 °C. (**d**) Comparison of the UTS at different thermal exposure temperatures.

Figure 2 shows the broken line diagram of the tensile properties of TC25G alloy after thermal exposure temperatures of 550 °C, 600 °C, and 650 °C with different times (0 h was the heat-treated state). It can be seen that after thermal exposure at 550 °C, 600 °C, and 650 °C, the variation trend of tensile properties at room temperature of alloy was inconsistent. When the thermal exposure temperatures were 550 °C and 600 °C, the ultimate tensile strength (UTS) and yield strength (YS) of alloy decreased firstly and then increased with the extension of thermal exposure time, and the reduction of area (RA) increased firstly and then decreased, while the variation trend of elongation (El) had little fluctuation. When the thermal exposure temperature was 650 °C, the UTS and YS of alloy decreased gradually at 0–5 h, began to increase at 5–100 h, but then decreased at 100–500 h. RA increased firstly, then decreased, and finally increased with the extension of thermal exposure time. In addition, with the extension of thermal exposure time at different thermal exposure temperatures, the change of RA was more significant than that of El, which indicated that RA was more sensitive to the change of thermal exposure time. It is worth noting that the UTS of alloy exposed at 650 °C was lower than that of heat-treated alloy.

When the thermal exposure temperature was 550 °C, as seen in Figure 2a, the UTS and YS of alloy were the lowest when thermal exposure time was 10 h, which decreased by 111 MPa and 92 MPa, respectively, compared to the heat-treated alloy. With the extension of thermal exposure time, the UTS and YS of alloy gradually increased and reached the maximum value when the thermal exposure time was 500 h, which increased by 27 MPa and 58 MPa, respectively, compared with heat-treated alloy. When the thermal exposure temperature was 600 °C, as seen in Figure 2b, the UTS and YS of alloy were the lowest when thermal exposure time was 10 h, which decreased by 109 MPa and 35 MPa, respectively, compared to the heat-treated alloy. With the extension of thermal exposure time, the UTS and YS of alloy gradually increased and reached the maximum value when thermal exposure time was 500 h, which increased by 33 MPa and 63 MPa, respectively, compared to the heat-treated alloy. When the thermal exposure temperature increased to 650 °C, as seen in Figure 2c, the UTS and YS of alloy were the lowest when the thermal exposure time was 5 h, which decreased by 86 MPa and 39 MPa, respectively, compared to the heat-treated alloy. With the extension of thermal exposure time, the UTS and YS of alloy gradually increased and reached the maximum values when thermal exposure time was 100 h. When the thermal exposure time was extended to 500 h, the UTS and YS of alloy began to decrease gradually, namely, by 58 MPa and 15 MPa, respectively, compared with heat-treated alloy. Interestingly, the average UTS of alloy exposed at 600 °C was always higher than that of alloy exposed to 550 °C at the same thermal exposure time, as shown in Figure 2d.

### 3.2. Microstructure

**Figure 3:**
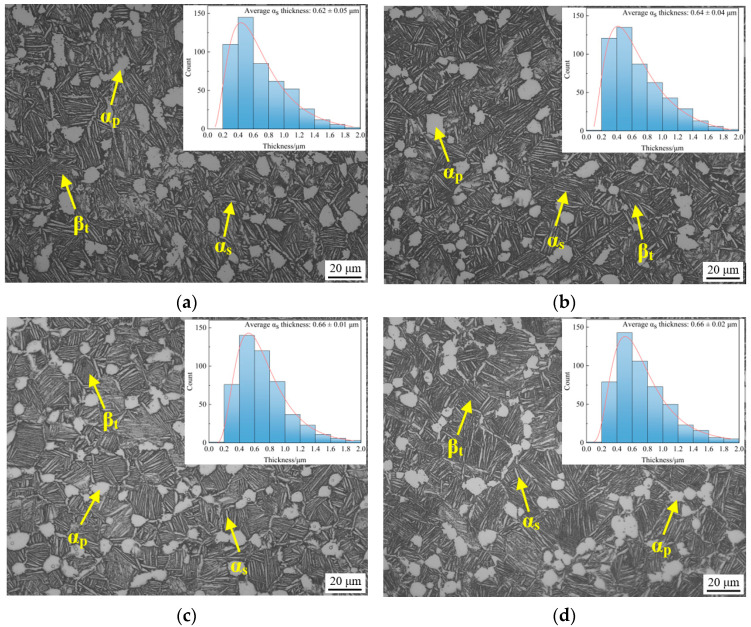
OM microstructure of TC25G alloy at different thermal exposure conditions: 550 °C for (**a**) 10 h and (**b**) 500 h; 600 °C for (**c**) 500 h; 650 °C for (**d**) 500 h.

Figure 3 shows the OM microstructure of TC25G alloy after thermal exposure at 550 °C and 650 °C for 100 h and 500 h, respectively. It was found that the microstructure of alloy is not much different, and both are typical mixed microstructures composed of the α_p_ phase and β_t_. The β_t_ with the original grain profile is composed of clusters with different geometric orientations, each of which consists of the lamellar α_s_ phase with common orientations and spaced apart and the residual β phase between the α_s_ phase. Compared with before thermal exposure (Figure 1), there was little difference in the size and volume fraction of the α_p_ phase in the OM. This is understandable because thermal exposure is part of the over-aging process in the heat treatment of titanium alloy, and aging treatment does not affect the size and volume fraction of the α_p_ phase but only the decomposition of residual metastable β phase retained after solid solution cooling, which forms the aging α phase and aging β phase. Since the aging α phase precipitated from the residual β phase is very small, they were both shown as gray with the residual β phase under the OM. Therefore, the size, morphology, and volume fraction of the aging α phase could not be distinguished by the OM. Image processing software of Image-pro Plus was used to calculate the average thickness of the α_s_ phase by measuring at least 500 intervals, and it was found that the thickness of the α_s_ phase also did not increase significantly, because the lamellar thickness of the α_s_ phase only depended on the cooling rate during the solution cooling process. Therefore, it is considered that the influence of thermal exposure temperature and time on the α_p_ phase and β_t_ in TC25G alloy is almost negligible.

**Figure 4:**
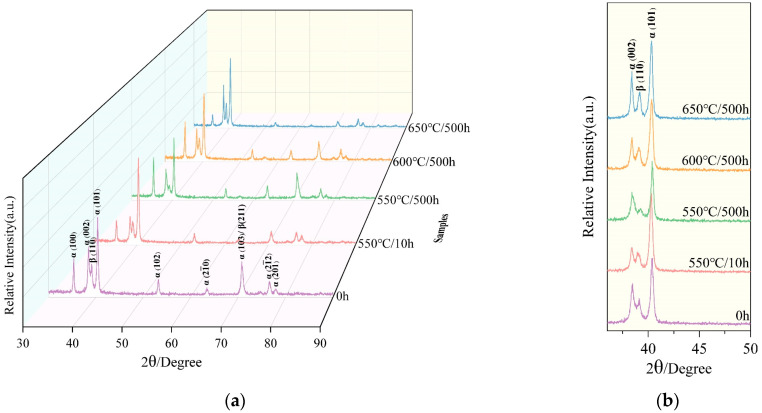
XRD images of TC25G alloy: (**a**) XRD image of samples after different thermal exposure conditions; (**b**) magnification of (**a**).

According to some literatures [25,26], the change of mechanical properties of alloy may be caused by the decomposition of the residual β phase and the precipitation of the α_2_ phase and silicide during the process of thermal exposure. Because of the small size of precipitates in TC25G alloy, it cannot be observed effectively under the OM. In order to further determine the type and content of precipitates, XRD analysis was conducted, as shown in Figure 4. It can be observed from the images that the intensity of the α (101) diffraction peak was the strongest among all peaks, and the intensity of the α phase diffraction peak was significantly higher than that of the β phase, indicating that the volume fraction of the α phase is greater than that of β phase. When the thermal exposure temperature was 550 °C, the diffraction peak of β (110) decreased because a large amount of aging α phase precipitated from the β phase, so the volume fraction of the β phase decreased. When thermal exposure conditions were 600 °C/500 h and 650 °C/500 h, the diffraction peak intensity of β (110) rose. This is because at higher temperature thermal exposure, the undercooling degree of β phase transformation into the α phase was lower, the nucleation rate of the aging α phase decreased, and the aging α phase tended towards coarsening and growing. The decrease of the aging α phase volume fraction led to the increase of the β phase volume fraction. Further observation showed that even at 650 °C/500 h, there were only diffraction peaks of the α phase and β phase in alloy, but not those of the α_2_ phase and silicide. This is because the content of the α_2_ phase and silicide generated in alloy during thermal exposure was less. In the XRD test, the minimum value required to produce a diffraction peak was not reached (the lower limit of the content is generally about 5%). Although there was little and tiny precipitate in the process of thermal exposure, the mechanical properties of alloy changed obviously, mainly due to the synergistic effect of the size and content of precipitate. In order to clarify the distribution of precipitates in TC25G alloy, TEM analysis was conducted.

## 4. Discussion

### 4.1. Heat Treatment

**Figure 5:**
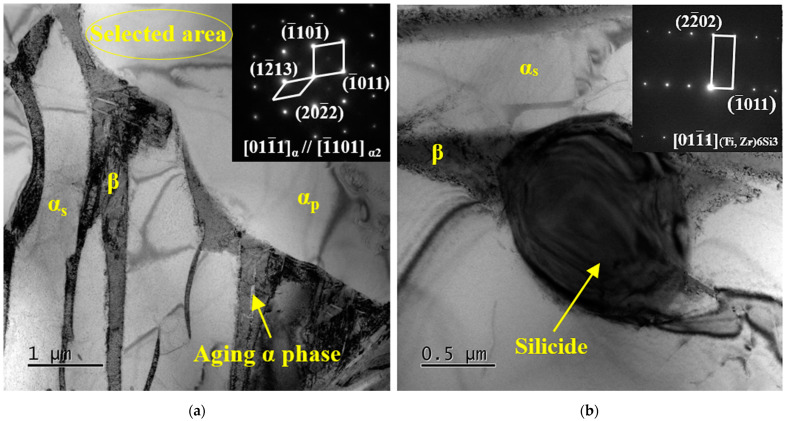
BF TEM images of TC25G alloy after heat treatment: (**a**) microstructure and SAED pattern of α_p_ phase; (**b**) distribution and morphology of silicide and its SAED pattern.

Figure 5 shows the TEM bright field (BF) images of TC25G after heat treatment. It can be observed from Figure 5a that during the aging process, the residual metastable β phase retained after solid solution cooling decomposed into a small number of nanoscale acicular aging α phase with a thickness of about 20 nm. In the process of residual metastable β phase decomposition, silicide precipitates at the α/β phase boundary can also be seen, with a small size of 0.9 μm in the short axis and 1.1 μm in the long axis, as shown in Figure 5b. This is because Si atoms are mainly solvable in the β phase and slightly solvable in the α phase. Due to the high concentration of Si atoms in the residual β phase, the concentration gradient of Si atoms in the α phase and β phase makes the α/β phase boundary not only provide a higher driving force for the nucleation of silicide, but it also accelerates the diffusion rate of Si atoms, so the α/β phase boundary is conducive to silicide precipitation. Silicide is identified as a hexagonal S2 type (Ti, Zr)_6_Si_3_ phase with a P6/mmm point group and lattice constants of a = b = 0.701 nm and c = 0.368 nm by calibration of selected area electron diffraction (SAED) [27]. At the same time, a very weak superlattice electron diffraction pattern can be seen in the selected area of the α_p_ phase in Figure 5a, indicating that after heat treatment, a small amount of the ordered α_2_ phase precipitates on the α_p_ phase. Due to the short aging temperature, the α_2_ phase has no time to grow, so its size is small and cannot be clearly observed in the TEM field.

### 4.2. Thermal Exposure at 550 °C

**Figure 6:**
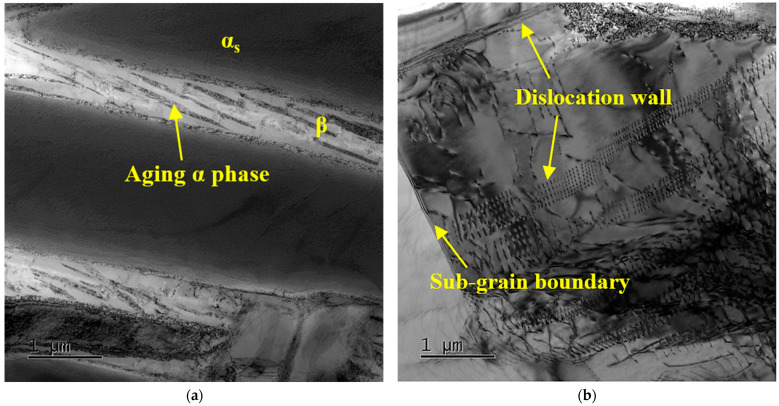
BF TEM images of TC25G alloy after thermal exposure at 550 °C for 10 h: (**a**) microstructure; (**b**) dislocation wall and sub-grain boundary in the α_p_ phase.

Figure 6 shows the TEM BF images of TC25G after thermal exposure at 550 °C for 10 h. Compared with heat-treated alloy, a large amount of the aging α phase precipitated on the residual β phase, as showed in Figure 6a, which is consistent with the XRD results in Figure 4. The volume fraction of the residual β phase decreased significantly, while the corresponding aging α phase increased obviously. It was proved that the thermal exposure process is beneficial to decomposition of the residual β phase because of the limited slip system of the HCP structure and the size constraint of the aging α phase, leading to the strength of the aging α phase being higher than the β phase, so it is difficult to generate plastic deformation. However, at the same time, a large number of regularly arranged dislocation walls were observed in the α_p_ phase, indicating that the microstructure recovered in a short time, resulting in a decrease in dislocation density and partial dislocation polygonization to form sub-grain boundaries, as shown in Figure 6b. Some literatures have analyzed and discussed the influence of precipitates and dislocations on the thermal stability of titanium alloy [28,29,30,31], and it is believed that the nucleation and growth of precipitates are the main reasons for the plasticity of titanium alloy after thermal exposure, and the recovery of dislocations will improve the plasticity of alloy to a certain extent. In the process of thermal exposure, the movement and redistribution of dislocations caused the encounter and cancelling of opposite sign dislocations on the same slip plane. The same sign dislocations rearranged to form the dislocations wall, which even led to polygonization and eventually formed sub-grains. This process is equivalent to annealing at low temperature, so the strength of alloy decreased and the plasticity increased, which is the softening and microstructure stabilization process of alloy.

**Figure 7:**
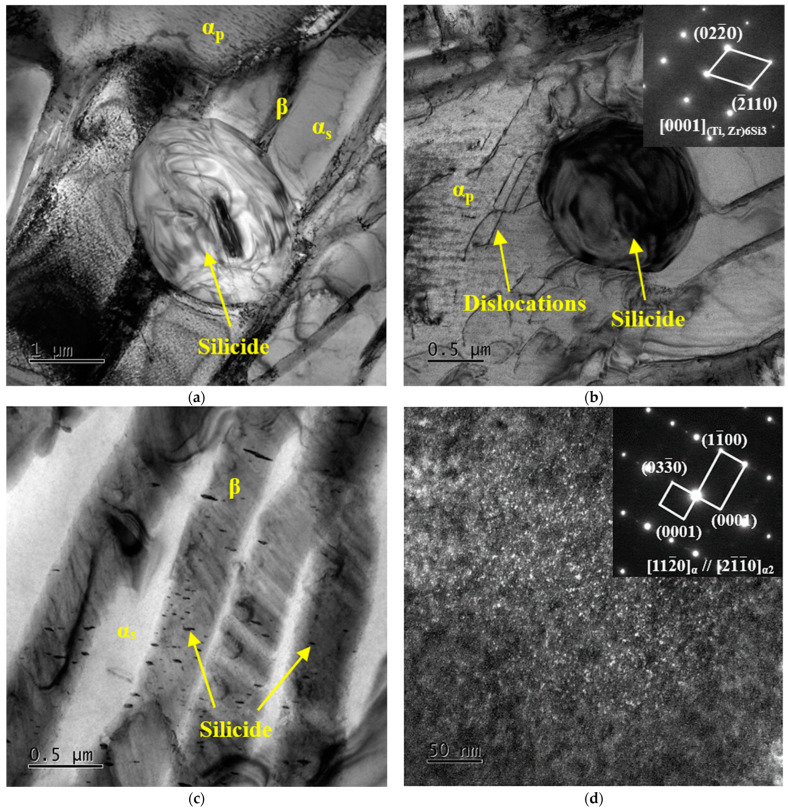
TEM images of TC25G alloy after thermal exposure at 550 °C for 500 h: BF micrograph showing distribution and size of silicide precipitated (**a**) at the α/β phase boundary, (**b**) at the dislocations of the α_p_ phase and its SAED, and (**c**) on the β phase; (**d**) DF micrograph of the α phase showing the α_2_ phase and super lattice diffraction spots from the α_2_ phase.

Figure 7 shows the TEM images of TC25G after thermal exposure at 550 °C for 500 h. After observation, the silicide precipitated at the α/β phase boundary was an ellipsoid with a short axis of 1.6 μm and a long axis of 2.3 μm, and its size increased significantly, as shown in Figure 7a. Generally speaking, due to the difference in solid solubility of the Si element between the α phase and β phase, the coarsening and growing phenomenon of silicide will result, along with the decomposition of the residual β phase during thermal exposure. Interestingly, with the extension of thermal exposure time, a small amount of spherical silicide gradually precipitated in the α_p_ phase and preferentially formed in the position of dislocation and other defects, as shown in Figure 7b. After SAED calibration, silicide was determined to be a hexagonal S2 type (Ti, Zr)_6_Si_3_ phase. It is worth noting that more ellipsoid precipitates with smaller sizes precipitated on the β phase, the size of which was about 20–200 nm in the long axis direction and 5–25 nm in the short axis direction, the average aspect ratio was approximately equal to 12.6, and the long axis direction was at an angle of 90° with the α/β phase boundary, as shown in Figure 7c. Because of its small size, it was impossible to calibrate the diffraction spots, but it could be determined as silicide according to its morphology. Figure 7d shows the dark field (DF) micrograph and SAED pattern on the α phase. The superlattice electron diffraction pattern shows that the reciprocal lattice of the α_2_ phase was evenly distributed in one half of the α phase matrix, which verified the coherent relationship between the α_2_ phase and α phase. The small bright spots are α_2_ phase particles, and a large number of ordered α_2_ phase precipitated from the α phase matrix. The α_2_ phase is highly dispersed, with an extremely fine average grain size (<1 nm) and close grain spacing. It has a certain dispersion strengthening effect, but the strengthening effect is small. In general, when thermal exposure temperature is 550 °C and the time is 10–500 h, the precipitation of much silicide and the α_2_ phase is the main reason for the increase of strength and the decrease of plasticity of alloy.

### 4.3. Thermal Exposure at 600 °C

**Figure 8:**
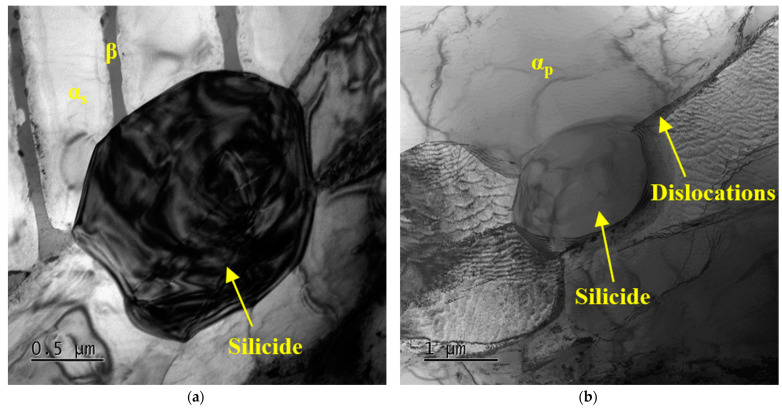

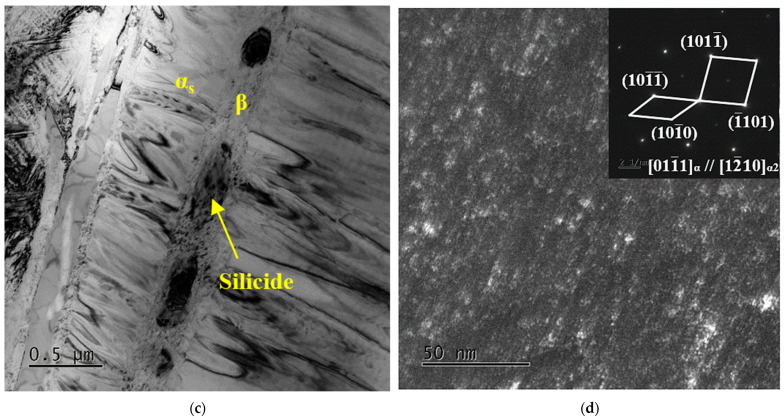
TEM images of TC25G alloy after thermal exposure at 600 °C for 500 h: BF micrograph showing distribution and size of silicide precipitated (**a**) at the α/β phase boundary, (**b**) at the dislocations of the α_p_ phase, and (**c**) on the β phase; (**d**) DF micrograph of the α phase showing the α_2_ phase and super lattice diffraction spots from the α_2_ phase.

Figure 8 shows the TEM images of alloy after thermal exposure at 600 °C for 500 h. As the decomposition rate of residual β phase decreased, the size of silicide precipitated at the α/β phase boundary did not grow significantly, as shown in Figure 8a. Silicide precipitated at the dislocation of the α_p_ phase grew from spherical to ellipsoidal, and its size increased significantly, as shown in Figure 8b. Silicide precipitated on the β phase coarsened and grew obviously, with the dimension range of 600 nm in the long axis direction and the dimension range of 200 nm in the short axis direction; the aspect ratio was approximately equal to 3, and the size of both the long and short axis increased to some extent, as shown in Figure 8c. Because these silicides are not coherent with the matrix, dislocations cannot pass through silicide during stretching but interact with them, resulting in stress concentration within the grains and at the phase boundaries. At the same time, the spherical α_2_ phase are also observed in α phase, with the average size growing to about 1 nm and the number slightly decreasing, as shown in Figure 8d. Because the nucleation of the α_2_ phase depends on the degree of undercooling, when thermal exposure temperature increases, the lower degree of undercooling reduces the nucleation rate of the α_2_ phase, thus providing more room for growth of the α_2_ phase. In addition, the growth of the α_2_ phase mainly depends on the diffusion rate of Al atoms in the α phase. The diffusion of Al atoms is a thermal activation process, which is greatly affected by temperature. High temperature has a higher driving force on the diffusion of Al atoms. This is consistent with the results of an earlier study by Radecka et al. on the near-alpha titanium alloy IMI834 [32]. The precipitation and growth of the α_2_ phase requires a certain thermal exposure temperature and time. The influence of ordered α_2_ on the mechanical properties of alloy is mainly due to the existence of the α_2_ phase, which changes or affects the motion state of dislocations. Since the α_2_ phase is a brittle phase, dislocations through the α_2_ phase mainly generated a cutting mechanism, which plays a certain role in hindering the movement of dislocations, and the dispersion strengthening effect is significant. Generally, when thermal exposure temperature is 600 °C and time is 10–500 h, the size increase of the α_2_ phase and silicide is the main reason for the strength increase and plasticity decrease of alloy.

### 4.4. Thermal Exposure at 650 °C

**Figure 9:**
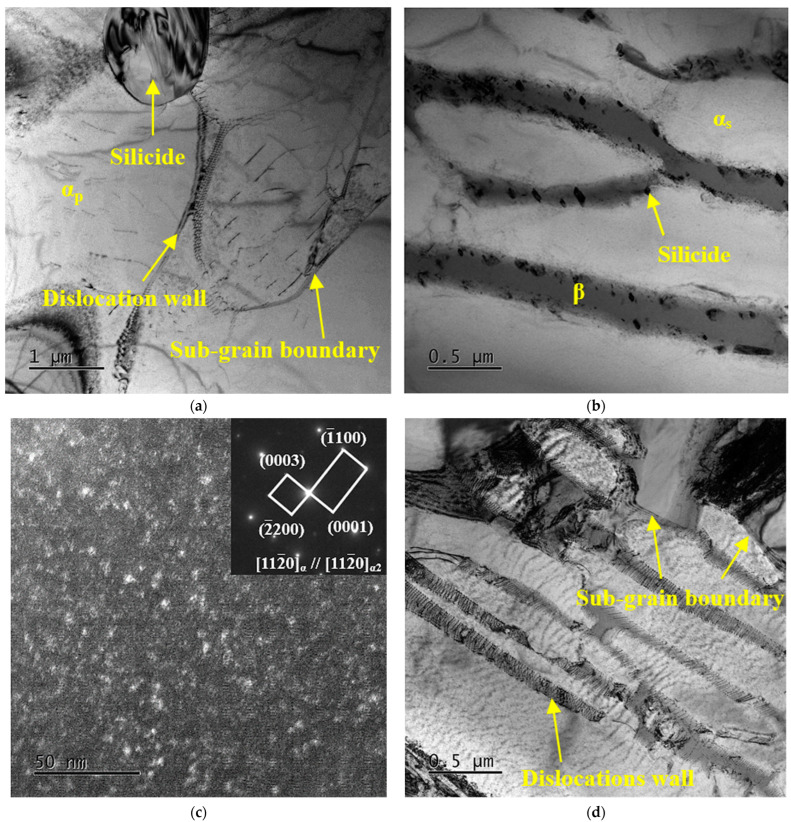
TEM images of TC25G alloy after thermal exposure at 650 °C for 100 h: BF micrograph showing distribution and size of silicide precipitated (**a**) at the dislocation of the α_p_ phase and (**b**) on the β phase; (**c**) DF micrograph of the α phase showing the α_2_ phase and super lattice diffraction spots from the α_2_ phase; (**d**) dislocation wall and sub-grain boundary in the α_p_ phase.

Figure 9 shows the TEM images of TC25G after thermal exposure at 650 °C/100 h. After a lot of observation, the size and quantity of silicide precipitated at the dislocation of the α_p_ phase and on β phase were basically stable, as shown in Figure 9a,b. Moreover, the average size of the α_2_ phase within the α phase grew to about 3 nm, as shown in Figure 9c. The UTS of alloys after thermal exposure at 650 °C is always lower than that of heat-treated titanium alloy. This is because the variation trend of alloy strength is the balance between the enhancement of the dispersion strengthening effect caused by the second phase precipitation and the weakening of the solid solution strengthening effect caused by the reduction of the solid solution atom content in the matrix. Specifically, the precipitation of silicide and the α_2_ phase can inhibit dislocation slip and improve the strength, which can also lead to the decrease in content of solid solution Si atoms and Al atoms in the matrix. When the dispersion strengthening effect caused by the growth of the α_2_ phase was offset by the decrease of the solid solution strengthening effect caused by the decrease of the solid solution Al atom content, the strengthening effect was weakened. At the same time, thermal exposure at 650 °C intensified the recovery of dislocation in the alloy, and the dislocation was more likely to elicit polygonization to form sub-grains, as shown in Figure 9a,d. However, in the time range of 5–100 h, the strength of alloy still showed an increasing trend, mainly because the weakening rate of solution strengthening is lower than the growth rate of dispersion strengthening.

**Figure 10:**
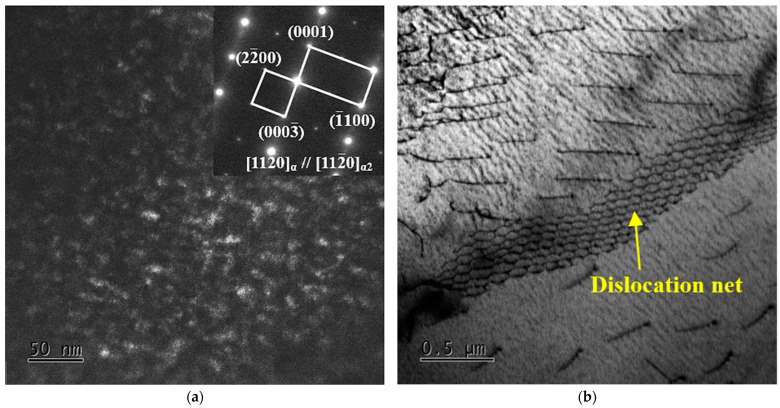
TEM images of TC25G alloy after thermal exposure at 650 °C for 500 h: (**a**) DF micrograph of the α phase showing the α_2_ phase and super lattice diffraction spots from the α_2_ phase. (**b**) Dislocation–particle interaction.

Figure 10 shows the TEM images of TC25G after thermal exposure at 650 °C for 500 h. A very strong superlattice electron diffraction pattern can be observed in Figure 10a. The average size of the α_2_ phase in the α phase is about 6 nm, and the grain spacing increases. When the thermal exposure temperature is 650 °C, the nucleation rate of the α_2_ phase is lower, but the growth rate of the α_2_ phase is accelerated. When the thermal exposure time is 500 h, Ostwald ripening of the α_2_ phase occurred, and the smaller α_2_ phase dissolved and disappeared, while the larger α_2_ phase coarsened and grew rapidly, and the density decreased obviously. This result is consistent with previous research results [33]; the higher the thermal exposure temperature, the larger the size of the α_2_ phase. However, this does not imply that the growth of the α_2_ phase is infinite. In fact, at a certain thermal exposure temperature, the growth rate of the whole precipitation process becomes slower and slower. It has been shown [30] that when the α_2_ phase is completely precipitated, its size will not change even if thermal exposure time is further prolonged. Due to the limitation of experimental time, the size of the α_2_ phase continues to grow and does not reach a stable state. However, the strength of the alloy decreases obviously after thermal exposure at 650 °C/100 h. According to the relationship between the critical decomposition shear stress and the particle size of the second phase, it was proven that 3 nm is the critical size of α_2_ phase. When the size of the α_2_ phase exceeds the critical value, the grain spacing will increase to a certain size. The mechanism of action between the α_2_ phase and dislocations changes from a cutting mechanism to a by-pass mechanism (Orowan mechanism), as shown in Figure 10b. This phenomenon is consistent with the results of Zhang et al. [17]. Although the strength of alloy decreases, the plasticity is obviously improved.

## 5. Conclusions

Thermal exposure experiments were carried out at 550 °C, 600 °C, and 650 °C to study the effects of temperature and time on the microstructure and mechanical properties of TC25G alloy. The results show the following:

(1) When thermal exposure temperature was 550 °C, the UTS and YS decreased firstly and then increased with the extension of thermal exposure time. When the time range was 0–10 h, due to the polygonization of dislocations and the formation of a sub-grain boundary, and while the strengthening effect of precipitation was weak, the strength of the alloy showed a downward trend. When the time range was 10–500 h, the precipitation of silicide precipitated at the dislocation of the α_p_ phase and on the β phase, and the precipitation of the α_2_ phase precipitated from the α phase also played a significant role in the dispersion strengthening effect.

(2) When the thermal exposure temperature was 600 °C, the UTS and YS decreased firstly and then increased with the extension of thermal exposure time. When the time range was 10–500 h, the increased size of silicide precipitated at the dislocation of the α_p_ phase and on the β phase, and the α_2_ phase precipitated from the α phase together enhanced the dispersion strengthening effect of the alloy, and the UTS and YS of the alloy reached 1140 MPa and 1042 MPa at 600 °C/500 h, respectively. At the same time, the UTS of the alloy after thermal exposure at 600 °C was always higher than that of the alloy after thermal exposure at 550 °C.

(3) When the thermal exposure temperature was 650 °C and the time range was 0–500 h, the concentration of Al atoms in the matrix decreased with the increase of the α_2_ phase size, and the precipitation strengthening effect was not enough to compensate for the decrease of the solid solution strengthening effect; moreover, the dislocation was more likely to polygonise and form sub-grains, so the strength of the alloy was always lower than that of the heat-treated alloy. However, the decreasing rate of solution strengthening was less than the increasing rate of dispersion strengthening, and the strength of the alloy still showed an increasing trend within 5–100 h. When the time range was 100–500 h, the size of the α_2_ phase increased from the critical value of 3 nm to 6 nm, and the interaction between the α_2_ phase and dislocations changed from the cutting mechanism to the by-pass mechanism, with, finally, sharply decreased strength and greatly improved plasticity.

## Data Availability

No applicable.
